# Biogenic Synthesis of Silver Nanoparticles Using *Phagnalon niveum* and Its In Vivo Anti-Diabetic Effect against Alloxan-Induced Diabetic Wistar Rats

**DOI:** 10.3390/nano12050830

**Published:** 2022-03-01

**Authors:** Muhammad Nisar Ul Haq, Ghulam Mujtaba Shah, Alia Gul, Ahmed Ibrahim Foudah, Mohammad Hamed Alqarni, Hasan Soliman Yusufoglu, Masroor Hussain, Huda Mohammed Alkreathy, Ihsan Ullah, Amir Muhammad Khan, Shahid Jamil, Mushtaq Ahmed, Rahmat Ali Khan

**Affiliations:** 1 Department of Botany, Hazara University Mansehra, Mansehra 21300, Pakistan; mnhq1987@gmail.com (M.N.U.H.); aliagulbotanist@gmail.com (A.G.); 2 Department of Pharmacognosy, College of Pharmacy, Prince Sattam Bin Abdulaziz University, Al-Kharj 11942, Saudi Arabia; a.foudah@psau.edu.sa (A.I.F.); m.alqarni@psau.edu.sa (M.H.A.); 3 Department of Pharmacognosy & Pharmaceutical Chemistry, College of Dentistry & Pharmacy, Buraydah Private Colleges, Buraydah 51418, Saudi Arabia; hasan.yusuf@bpc.edu.sa; 4 Department of Biotechnology, University of Science and Technology Bannu, Bannu 28100, Pakistan; masroorktk@gmail.com (M.H.); shahidberg@uop.edu.pk (S.J.); mushtaq121@yahoo.com (M.A.); 5 Department of Pharmacology, Faculty of Medicine, King Abdulaziz University, Jeddah 21589, Saudi Arabia; halkreathy@gmail.com; 6 Department of Botany, University of Science and Technology Bannu, Bannu 28100, Pakistan; iukwazir@gmail.com; 7 Department of Botany, University of Mianwali, Mianwali 42200, Pakistan; khanamir62@yahoo.com

**Keywords:** *Phagnalon niveum*, silver nanoparticles, diabetes mellitus, alloxan animal model, green nanomedicine

## Abstract

**Background**: Type-2 diabetes mellitus (T2DM) is a non-communicable, life-threatening syndrome that is present all over the world. The use of eco-friendly, cost-effective and green synthesised nanoparticles (NPs) as a medicinal therapy in the treatment of T2DM is an attractive option. Aim: The present study aimed to evaluate the anti-diabetic potential of the phyto-synthesised silver nanoparticles (AgNPs) obtained from *Phagnalon niveum* plant methanolic extract. **Methods**: The green synthesised AgNPs made from *Phagnalon niveum* plant methanolic extract were analysed by Ultraviolet-Visible (UV-Vis) spectroscopy, and the functional groups involved in the reduction of the silver ions (Ag^+^) were characterised by Fourier Transform Infrared (FTIR) spectroscopy. The size and crystallinity were assessed via X-ray Diffraction (XRD). The morphology of AgNPs was confirmed using Scanning Electron Microscopy (SEM). The amount of silver (Ag) was estimated via energy dispersive X-ray (EDX) analysis. An intraperitoneal injection of 200 mg alloxan per kg albino Wistar rats’ body weight, at eight weeks old and weighing 140–150 g, was used to induce diabetes mellitus (N = 25; n = 5/group). Group C: untreated normal control rats that only received distilled water, group DAC: diabetic control rats that received alloxan 200 mg/Kg body weight, DG: diabetic rats treated with glibenclamide at 0.5 mg/kg body weight, DE: diabetic rats that received methanolic *P. niveum* extract at 10 mg/Kg body weight, and DAgNPs: diabetic rates that received AgNPs synthesised from *P. niveum* at 10 mg/kg body weight. The blood glucose levels were monitored on days 0, 7, and 14, while lipid, liver, and kidney profiles were checked after dissection at the end of treatment (day 21). On the final day of the period study (day 21), an oral glucose tolerance test was carried out by administering orally 2 g/kg body weight of glucose to the respective groups, and the blood glucose level was checked. A fasting glucose level was measured using a glucometer. Urine samples were collected from each animal and analysed using lab-made assay kits for glucose, bilirubin, pH, leukocytes, and nitrite, among other factors. For statistical analyses, a one-way ANOVA and Dunnett’s test were applied. **Results**: The green-mediated synthesis of AgNPs using *P. niveum* methanolic extract produced spherical and mono-dispersed NPs with a size ranging from 12 to 28 nm (average: 21 nm). Importantly, a significant reduction of blood glucose levels and an increase in body weight, as well as a remarkable improvement in lipid, liver, and kidney profiles, were noticed. **Conclusions**: The biosynthesised AgNPs significantly improved the abnormalities in body weight, urine, and serum levels, indicating that it is a promising anti-diabetic agent.

## 1. Introduction

Nanotechnology has recently taken on a significant role in scientific research and development. The field of nanotechnology deals with the manufacture and development of nanoscale particles with a size range of 1–100 nanometres (nm), comparatively small compared to bulk materials [1]. There is a current focus on the creation of non-materials and particles for delivery purposes, with consideration given to the location of action, drug size, and duration of the drug delivery order [2].

Due to its usage in a variety of sectors, nanotechnology is recognised as interdisciplinary due to the fact that it may be used as a biological diagnostic tool as well as in manufacturing tools and biosensors [3,4]. Developing nanotechnology throughout that time period has dubbed the twenty-first century the “Nano Century” [5]. Chemical, physical, and green methods are all used to synthesise nanoparticles. The utilisation of biological plants, bacteria, and other organisms in nanoparticle production is becoming more prevalent these days [4]. High pressures and high temperatures are used in the physical technique to synthesise nanoparticles. The reducing and stabilizing agents used in the chemical technique are very hazardous and expensive. The green technique of nanoparticle synthesis uses plants and plant parts, such as stems, bark, leaves, roots, seeds, flowers, fruits, gum, and buds, that are nontoxic, environmentally friendly, and cheap [6].

Diabetes mellitus is a serious hazard to human health across the globe, given its associated consequences [5]. In the whole world, it is among the top five leading causes of death [7]. Diabetes mellitus is a collection of metabolic illnesses that develop as a result of hyperglycaemia and glucose intolerance (DM). There are two commonly recognised kinds of diabetes mellitus. Type-1 diabetes is characterised by insufficient insulin release from the pancreatic cells, whereas type-2 diabetes is characterised by the development of the body’s insulin resistance [8]. More than 90% of diabetics across the world have type-2 diabetes mellitus [9]. This population is growing at an alarming pace. Diabetes mellitus affects more than 280 million people in the United States, and the high incidence of the disease might lead to an additional 400 million people being diabetic by 2030 [10].

There is a significant frequency of diabetes mellitus in contemporary lifestyles due to a lack of physical exercise, obesity, ethnicity, advanced age, and genetic polymorphism [11,12]. Type-2 diabetics have insulin resistance in their bodies, which prevents their cells from taking glucose from the blood, resulting in hyperglycaemia or high blood glucose levels. Without treatment, persistent hyperglycaemia may cause metabolic disorders in a variety of organs, including the kidneys. These metabolic problems may be deadly. Even if one does not die, diabetes can still lead to secondary issues, such as kidney, heart, liver, and eye illness, as well as erectile dysfunction and blindness [13,14].

The main cause of diabetes is reactive oxygen species (ROS)-induced oxidative stress, which leads to the malfunctioning of cells, insulin resistance, and poor glucose tolerance. ROS are formed when glucose and fatty acids are overloaded due to overeating and inactivity [15]. Apoptotic cell death is a consequence of physiological and pathological stress on the pancreatic cells [16]. Researchers also found a link between oxidative stress and pancreatic cell death, as well as diabetes-related side effects. They claim that hyperglycaemia-induced diabetic problems are primarily caused by an imbalance between ROS and TORCH, which raises oxidative stress and causes apoptosis [17]. As a result, by decreasing ROS production, these diabetes problems may be successfully managed.

Changes in food and lifestyle, as well as the oral delivery of anti-diabetic medications, are critical in reducing ROS formation during diabetes therapy. Whether you have type-1 or type-2 diabetes, it is essential to keep blood glucose levels within the normal range (70–140 mg/dL). Finding a medicine that works well for treating diabetes is a typical challenge. In the treatment of diabetes, many anti-diabetic and hypoglycaemic medications, such as sulfonylureas and biguanides, have been launched. However, these treatments do not give long-term control over the blood glucose level. These medications are also less prevalent since their extended usage leads to toxicity and unintended side effects, such as gastrointestinal pain, hypoglycaemia, pancreatic degeneration, and liver damage in the body [18]. Finding novel hypoglycaemic medications and hypoglycaemic agents with fewer side effects and better effectiveness is a worthwhile endeavour, and this has been the focus of previous research.

**Figure 1:**
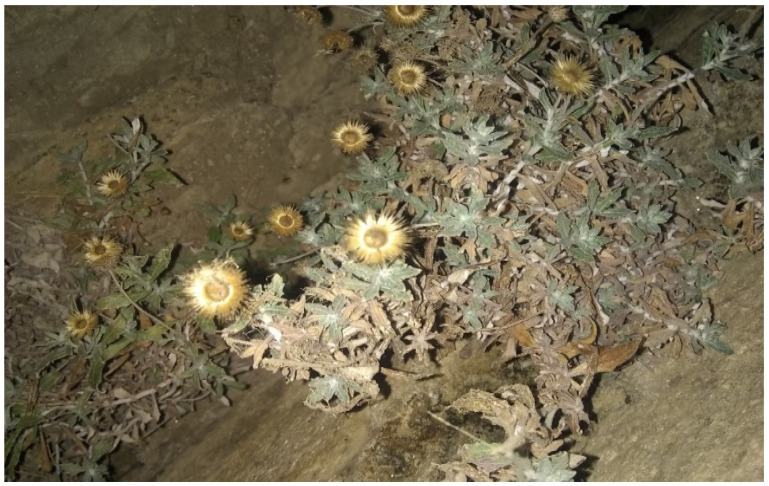
*Phagnalon niveum* Plant.

*Phagnalon niveum* is an Asteraceae family member with an upright or decumbent stem, 30–40 cm in height, with a few to an abundant number of developed branches, and a thick, snow-white cottony fleece covering the stem and the branches. There are many foliated branches on this plant. A wide range of leaf shapes and sizes are seen in this species, with the most common being lanceolate (8–35 mm) with an undulate or shallowly lobed margin, and the least common being oblanceolate (dentate or shallowly lobed), all having a sessile base. Since ancient times, the peduncles have been upright, measuring 20–70 mm and covered in a white cottony fleece that bears a single capitulum. It has multiflorous capitula that are 8–12 mm wide. Its external phyllaries are subulate to barely lanceolate and measure 2–2.5 mm in length. Its centre phyllaries are 3–3.5 mm wide and measure 0.5 mm in length. The latter are subulate to barely lanceolate and gradually narrow into an acicular point with whole edges. Their pinnacles are subulate to barely lanceolate. The flowers have no preference for tubulose, and are about 5–6 mm long, tube glabrous, with projections slightly adjusted; they are bristly at the ends, and have tube florets with no preference. Sub-exserted anthers contain completely ecaudate bases, including subincluded, papillose style, bifid and adjusted disgrace markings. Insufficiently bristly Pappus setae, about one millimetre long, barbellate almost all the way to the base [19]. *Phagnalon niveum* (Figure 1) is traditionally used in the treatment of diabetes mellitus and has not been investigated pharmacologically. Therefore, a comparative analysis of the anti-diabetic activity of the plant extract and green synthesised NPs was arranged.

## 2. Material and Methods

### 2.1. Chemicals and Instruments

We used methanol, ddH_2_O, AgNO_3_, NaOH, Aqua regia, DMSO, formalin, normal saline solution, gastric cannula, disposable syringes, a dissecting board, a dissecting box, diethyl ether, and cotton.

UV-Vis spectroscopy (Specord 200 plus Jena, Germany)

FTIR spectroscopy (Thermo Fisher Scientific, Waltham, MA, USA, Nicolet iS50 FT-IR)

XRD (JDX 3532, JEOL, Tokyo, Japan)

SEM (JSM 5910 JEOL, Japan)

EDX (INCA 200, Oxford, UK)

### 2.2. Plant Collection

During the months of April and May 2019, *Phagnalon niveum* Edgew (Figure 1) was collected in the Talash District, Dir Lower Khyber Pakhtunkhwa, Pakistan. The plant was properly identified by taxonomist Prof. Dr. Ghulam Mujtaba Shah and a voucher specimen (PN-134) has been submitted to the herbarium of the Department of Botany at Hazara University, Mansehra, for future reference. After identification, the plant was properly washed with water to remove dust particles and was shed-dried for 15 days at room temperature according to the previous investigations [20].

### 2.3. Extract Preparations of Phagnalon niveum Edgew

After fifteen days, the plant was ground to a fine powder and 100 g of powder was soaked in 300 mL of methanol and shaken vigorously. After 7 days, the filtrated Whatman No. 1 filter paper was dried with a rotary evaporator to obtain a thick, viscous, gummy crud extract. The crude extract was weighed and stored at 4 °C for further activities.

### 2.4. Phytochemical Screening

According to previously published protocols, numerous phytochemical screening tests were conducted [21,22]. The following tests were used in order to identify the phytochemicals.

### 2.5. Test

#### 2.5.1. Test for Alkaloids

Using Molisch’s Carbohydrate Test, two drops of an alcoholic naphthol solution are added to 2 mM of filtrate, and the mixture is agitated vigorously before 1 mM of concentrated sulphuric acid is slowly poured along the tube’s sides and left to stand for one min. Carbohydrates are indicated by a violet ring [21,22].

#### 2.5.2. Test for Glycosides

For the determination of glycosides, the Bontrager test was used. This involves hydrolysing 50 mg of extract in a water bath for 2 h with strong hydrochloric acid before filtering. A chloroform layer is separated, and a 10% ammonia solution is added to 2 mL of filtered hydrolysate. The chloroform layer is then added to the 10% ammonia solution and shaken. Glycosides are indicated by a pink tint [21,22].

#### 2.5.3. Test for Saponins

The saponins in 50 mg of extract are detected by diluting it with distilled water and making it into a 20 mL solution. For 15 min, the suspension is shaken vigorously in a graduated cylinder. Saponins are detected by looking for a 2 cm layer of foam [21,22].

#### 2.5.4. Testing for Proteins and Amino Acids

Here, 100 mg of extract is dissolved in 10 mL of distilled water and filtered through Whatman No. 1 filter paper [21,22].

##### Protein Biuret Test

A drop of 2% copper sulphate solution is added to a 2 mL aliquot of the filtrate. To this, 1 mL of 95% ethanol is added, and then an excess of potassium hydroxide pellets is poured over the top. The presence of proteins is indicated by the ethanolic layer’s pink colour [21,22].

##### Test for Amino Acids

The amino acid test using ninhydrin was performed. To 2 mL of aqueous filtrate, add two drops of ninhydrin solution (10 mg ninhydrin in 200 mL of acetone). The presence of amino acids is indicated by a distinctive purple hue [21,22].

#### 2.5.5. Test for Phytosterols

A biochemical assay for phytosterols involves dissolving a 50 mg extract in 2 millilitres of acetic anhydride. Add a few drops of concentrated sulfuric acid around the tube’s rim and stir carefully until the acid dissolves completely. Phytosterols are visible as a wide range of colour changes [21,22].

#### 2.5.6. Test for Phenols

Phenolic compounds are detected using the ferric chloride test, which involves dissolving 50 mg of the extract in 5 mL of distilled water. A few drops of a neutral ferric chloride solution (which contains 5% ferric chloride) are added to this. Phenolic substances are indicated by a dark-green hue [21,22].

#### 2.5.7. Test for Flavonoids

Using an alkaline reagent solution, an extract is treated with a 10% solution of ammonium hydroxide to look for flavonoids. Flavonoids are shown by a yellow glow [21,22].

#### 2.5.8. Test for Terpenoids

An extract of the plant is combined with 2 mL of chloroform (CHCl_3_) and 3 mL of concentrated H_2_SO_4_ to create a layer, and this mixture is put on the sample. The presence of terpenoids in the interface is indicated by a reddish-brown colouring [21,22].

#### 2.5.9. Test for Tannins

To find out whether there are any tannins present, use the following test: To 1 mM of plant extract, two millilitres of 5% ferric chloride is added. The presence of tannins is indicated by the formation of a dark-blue or greenish-black coloration [21,22].

#### 2.5.10. Test for Quinones

The extract was tested for quinones by adding 1 mL of concentrated sulphuric acid to 1 mL of extract. The presence of quinones is indicated by the formation of a red hue [21,22].

#### 2.5.11. Test for Coumarins

To test for coumarins, check for the presence of coumarins by performing the following test: 1 mL of the extract is mixed with 1 mL of 10% sodium hydroxide solution. The presence of coumarins is indicated by the formation of a yellow colour [21,22].

### 2.6. Synthesis of Silver Nanoparticles

The synthesis of silver nanoparticles was carried out using the method of Khan et al. (2018). After dissolving 100 mg of methanol crude in 50 mL of ddH2O, various amounts of extract (100 L, 300 L, 500 L, 800 L, 1 mL, 1.5 mL, 2 mL, 2.5 mL, and 3 mL) were placed in a 1 mmol solution of AgNO_3_ with various pH ranges (6, 7, 8, 9, 10, 11, and 12). At pH 11, the required nanoparticles are synthesised.

### 2.7. Characterisation of Silver Nanoparticles

Various characterisation techniques were used, such as UV-Vis, FTIR, XRD, SEM and EDS.

The surface plasma resonance (SPR) bonds of the plant-mediated silver nanoparticles were characterised using UV-Vis spectroscopy (Specord 200 plus Germany) in the range of 200–800 nm wavelength [23]. The surface and surface composition were analysed through FTIR spectroscopy (Nicolet iS50 FT-IR Thermo Fisher Scientific USA) in the mid-range of IR 4000–500 cm^−1^.

An X-ray diffraction technique was used to investigate the size and crystal structure of silver nanoparticles [23]. To check the morphology of silver nanoparticles, the SEM of the nanoparticles was performed by using a Scanning Electron Microscope (JSM 5910). To analyse the elemental composition of the analysed volume, EDX was performed on phyto-synthesised silver nanoparticles [24].

### 2.8. Induction of Diabetes

For this study, the Sengottaiyan et al. [24] protocol was used. All the experimental protocols for in vivo animal assays have been approved by the ethical committee. An intraperitoneal injection of 200 mg alloxan per kg of albino Wistar rats’ body weight, at eight weeks old and weighing 140–150 g, was used to induce diabetes mellitus (DM). If the blood glucose level rose to 220 mg/dL after 72 h, the animals were considered to be diabetic.

### 2.9. Acute Toxicity

AgNPs were tested for acute toxicity by administering doses of 10 and 20 mg per kg of body weight. During the 2 h period, any changes in neurology, physiology, or behaviour and fatality or lethality were recorded for up to 72 h with daily monitoring. The experiment was conducted by using 10 mg/kg AgNPs in accordance with a previously documented procedure [25].

### 2.10. Experimental Treatments in Animals and Ethics

The albino Wistar rates were purchased from the National Institute of Health (NIH) Sciences, Islamabad, Pakistan. They were fed with the NIH-recommended pellets of feed.

In a further step, the animals (n = 25) were divided into five groups, with five animals in each group, namely:

C: untreated normal rats (normal control, who received only distilled water);

DAC: diabetic control, with rats receiving alloxan 200 mg/kg body weight;

DG: diabetic rats treated with glibenclamide at 0.5 mg/kg body weight;

DE: diabetic rates treated with methanolic *P. niveum* extract at a dose of 10 mg/kg body weight;

DAgNPs: diabetic rats that received AgNPs synthesised from *P. niveum* at 10 mg/kg body weight.

After 21 days, the animals were sacrificed.

### 2.11. Oral Glucose Tolerance Test

The oral glucose tolerance test was performed on the final day of the period study (day 21), and the blood glucose level was checked periodically by collecting blood from the tail tip of each animal at 0, 30, 60, 90, and 120 min after orally administering 2 g/kg body weight of glucose to the respective groups. The fasting glucose level was also assessed using a glucometer (Accu-check Instant, Roche, Germany).

### 2.12. Serological Analysis

A retro-orbital puncture was used to obtain blood samples, which were then centrifuged at 1000 rpm for 15 min. The serum was then collected and kept cold (−80 °C) until needed. Alanine aminotransferase (ALT), alkaline phosphatase (ALP), bilirubin, total protein, bilirubin, creatinine, and high-density lipoprotein-cholesterol (HDL-c) were measured using the technique previously described [26,27,28,29].

### 2.13. Urine Analysis

For each animal, urine samples were collected and various levels were assessed, namely, leukocytes, nitrite, urobilinogen, protein, pH, blood, specific gravity, ketone, bilirubin, and glucose.

### 2.14. Statistical Analysis

All data from three independent experiments were expressed as mean SD, and the statistical significance between groups was determined using a one-way ANOVA followed by Dunnett’s test in Origin 18. At a *p*-value of 0.05, the data would be considered significant.

## 3. Results

### 3.1. Phytochemical Analysis

Biochemical screening results showed that phenols, proteins, tannins, flavonoids, alkaloids, quinones, sterols, carbohydrates, amino acids, terpenoids, and coumarins were present in the crude extract of *P. niveum*. However, the crude extract did not show any colour change for saponins and glycosides tests (Table 1).

### 3.2. Synthesis and Characterisation of Silver Nanoparticles

#### 3.2.1. Green Synthesis of AgNPs

**Figure 2:**
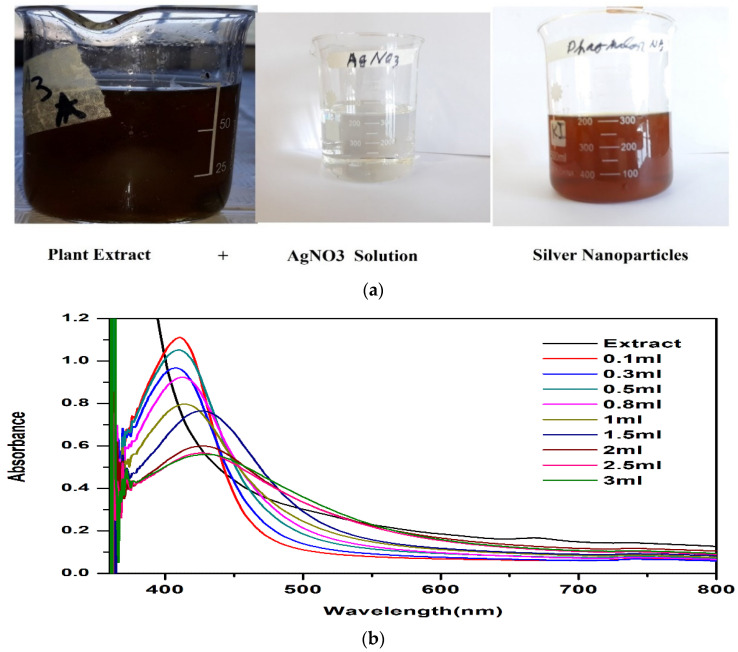
(**a**): Synthesis of AgNPs and colour change; (**b**): UV-Vis spectroscopic study of *P niveum* AgNPs.

Various amounts (0.1, 0.3, 0.5, 0.8, 1, 1.5, 2, 2.5, and 3 mL) of *P. niveum* extracts were mixed with a silver nitrate (AgNO_3_) solution (0.085g by weight and 1 mM solution) and the colour changed from yellowish to dark brown, which indicated the reduction of silver ions (Ag^+^) and the synthesis of NPs (Figure 2a).

#### 3.2.2. UV-Vis Spectroscopy

The synthesis of AgNPs was confirmed by using the UV-Vis spectroscopy technique. The mechanism of synthesis of AgNPs was explained by the surface plasmon resonance (SPR) effect (Figure 2b). The *P. niveum* plant extract displays a high number of phenols, proteins, tannins, flavonoids, alkaloids, quinones, sterols, carbohydrates, amino acids, terpenoids, and coumarins, which contribute to the formation of AgNPs. Similarly, the UV-Vis absorbance spectrum of AgNPs at various concentrations and at different time intervals revealed a maximum absorbance peak between 360 and 410 nm, with an intensity varying between 0.5 and 0.9 (Figure 2b). The difference in the wavelength was most likely due to the different sizes of AgNPs.

#### 3.2.3. Fourier-Transform Infrared Spectroscopy

**Figure 3:**
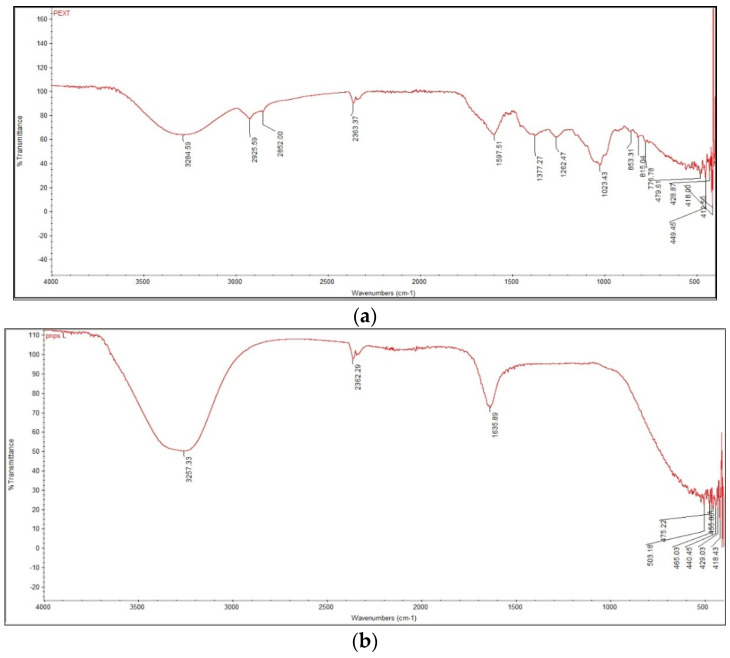
(**a**) FTIR spectra of *P. niveum* methanol extract. (**b**) FTIR spectra of AgNPs.

Figure 3a shows a major FTIR peak of the plant crude extract, observed at 3284.59 cm^−1^, which is assigned to the O–H stretching and bending bond. The O–H band gave rise to a broad peak, and this band verifies the presence of phenols. The peak at 2925 cm^−1^ is attributed to –CH2–(methylene) stretching, which gave rise to two C–H stretching bands, corresponding to symmetric and asymmetric modes of stretching. The peak at 2362.3 cm^−1^ shows O–H stretching. In Figure 3, the peaks at 1597 cm^−1^ show a C=O for the ring and the peaks at 1633 cm^−1^ show the conjugation of C=O with phenyl compounds. The peak at 1377.27 cm^−1^ refers to C–O–H bending, and vibrations are assigned to CH_3_. The peak at 1262.47 cm^−1^ shows strong C–O single bond stretching and vibrations that indicate the presence of phenols. The peak at 1023 cm^−1^ shows out of plan C–H bending and the vibration of the bend, which indicates the presence of an aromatic ring. The presence of this absorption in the IR region shows that the plant extract contains cyclic aromatic phenols, such as those found in flavonoids. Interestingly, the peaks observed in the IR spectrum of the synthesised AgNPs are shifted and decreased in intensity, which strongly suggests that plant biomolecules interact with AgNPs, most probably through their oxygen functionalities, which act as reducing and capping agents (Figure 3b).

#### 3.2.4. XRD Analysis

**Figure 4:**
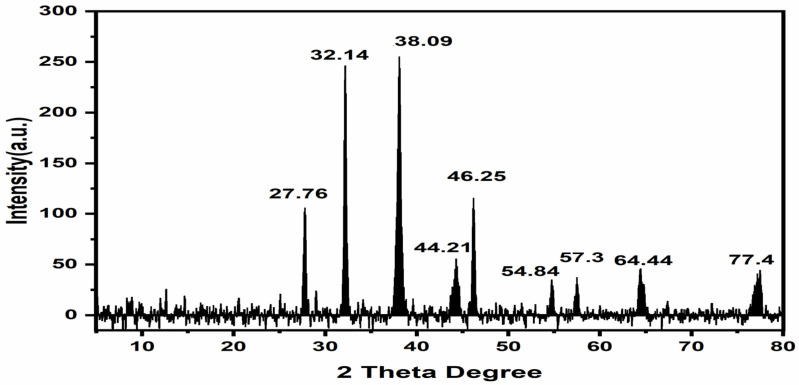
XRD pattern of *P niveum* AgNPs.

Then, we sought to confirm the crystalline nature of the biosynthesised PAgNPs with an XRD spectroscopic study. As shown in Figure 4, the XRD pattern shows a number of Bragg reflections at two values of 27.76°, 32.14°, 38.09°, 46.25°, 54.84°, 57.3°, 64.44°, and 77.4°, which correspond to the (111), (200), (200), (220), (311), (222), (220), and (311) sets of planes, respectively. These findings support the presence of face-centred cubic AgNPs. The lattice constant calculated for this structure was a 14 of 4.0855 A. The unassigned peaks might have resulted from the plant extract-dependent capping agent involved in the stabilisation of AgNPs, and the average size of the AgNPs was 21 nm.

#### 3.2.5. SEM Analysis

**Figure 5:**
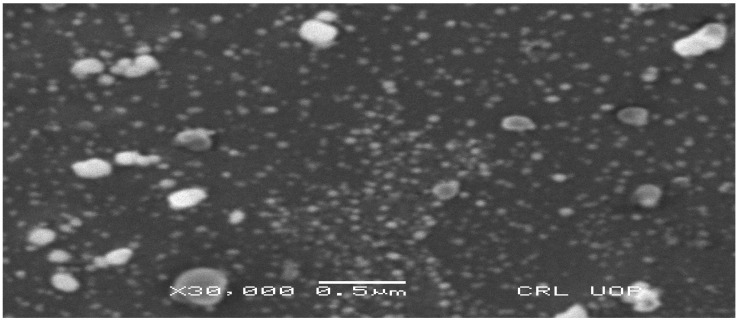
SEM analysis of *P. niveum* AgNPs.

To determine the shape of the green AgNPs, SEM analysis was performed using centrifuged powder of AgNPs. As shown in (Figure 5), they were spherical in shape, and did not agglomerate (even after 7 days).

#### 3.2.6. EDX Analysis

**Figure 6:**
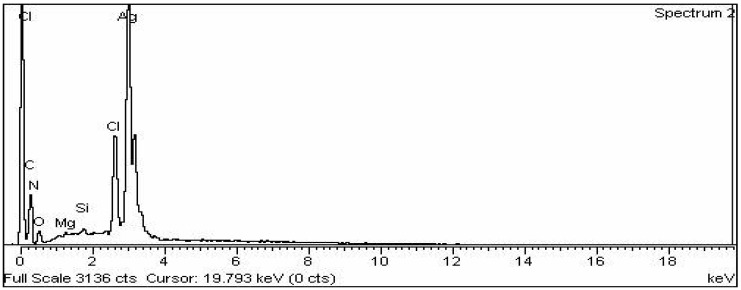
EDX analysis of *P. niveum* AgNPs.

The elemental configuration of the biosynthesised AgNPs was established using EDX, a detector connected to the SEM machine (Figure 6). The plotted graph exhibited a strong peak at 3 Kev, revealing the presence of Ag and confirming the existence of AgNPs. The Ag element accounted for 65.69% of the total composition. EDX analysis also revealed the presence of other elements, such as C, N, O, and Cl.

### 3.3. Acute Toxicity Test

In a short-term toxicity assessment, the methanol extract and green generated AgNPs were shown to be non-toxic. *P. niveum*-treated rats did not display any outward evidence of toxicity throughout the whole trial, and no mortality was reported. Alloxan-induced diabetic rats were treated with 10 mg/kg body weight of AgNPs, which was determined to be the best dose based on acute toxicity tests.

### 3.4. Blood Glucose Level

**Figure 7:**
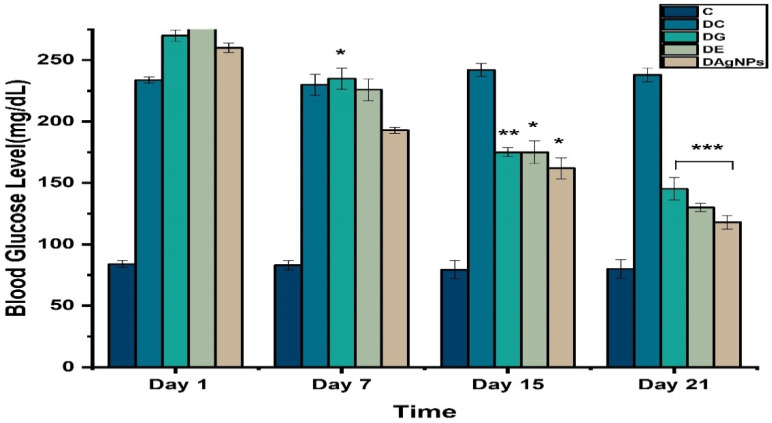
Effect of methanolic extract and synthesised *P niveum* AgNPs on blood glucose levels.

In order to assess the anti-hyperglycaemic impact of plant extract and phyto-synthesised AgNPs, the blood glucose levels of each group were measured over the duration of therapy (Figure 7). When compared to the diabetic control group, treatment groups (extract/AgNPs) had significantly lower blood glucose levels on days 1, 7, 14, and 21 (group DC). Compared to plant extract, AgNPs were shown to have a greater effect in lowering blood glucose levels. Groups treated with glibenclamide had substantially lower blood glucose levels compared to groups DC, DE, and DAgNPs throughout the course of therapy.

### 3.5. Oral Glucose Tolerance Test

**Figure 8:**
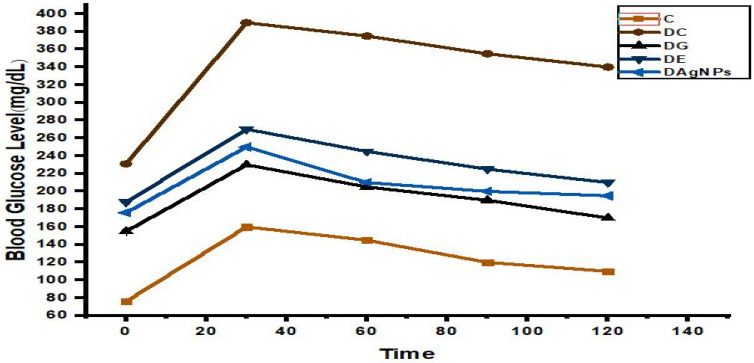
Effect of methanol extract and synthesised *P. niveum* AgNPs on oral glucose tolerance test.

*P. niveum* extract-mediated AgNP-treated diabetic rats (Figure 8) were compared to a standard treatment (glibenclamide 0.5 mg/kg) for their ability to reduce hyperglycaemic conditions in the blood after the administration of glucose orally. Compared to group DC, animals treated with phyto-synthesised AgNPs showed a considerable improvement, with decreasing blood glucose. For groups DG, DAgNPs, and DE, their blood glucose levels decreased over a duration of two hours. According to the results, phyto-synthesised AgNP administration improves blood glucose levels equivalent to glibenclamide-treated groups, according to the results. The diabetic rat model generated by alloxan has been extensively utilised in a number of studies.

### 3.6. Body Weight

**Figure 9:**
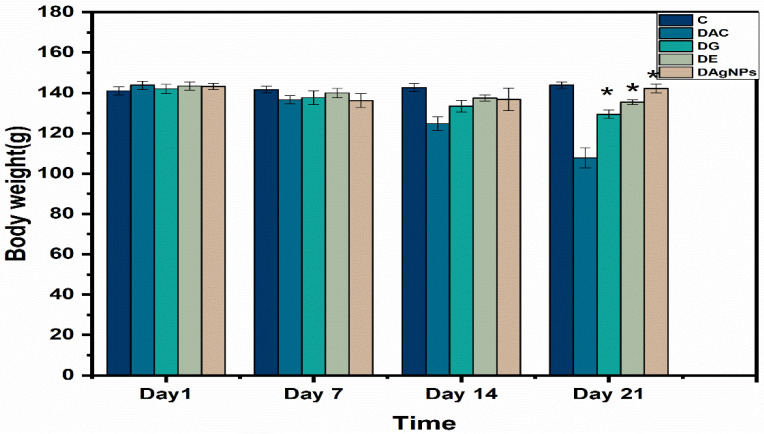
Effect of methanolic extract and synthesised *P. niveum* AgNPs on body weight.

When compared to diabetic rats, oral treatment of *P. niveum* extract and AgNPs at a dosage of 10 mg/kg body weight daily for 21 consecutive days significantly increased body weight (Figure 9). Compared to diabetic rats in group DC, the animals in groups DE and DAgNPs gained weight on day 21. However, as compared to the normal control group, the body weight of groups DE and DAgNPs rose considerably (*p* 0.01). Orally, there was no difference in body weight between the glibenclamide group DG and the equivalent control group C after the administration of 0.5 mg/kg body weight *P. niveum* extract orally (Figure 9).

### 3.7. Serological and Urine Parameters

**Figure 10:**
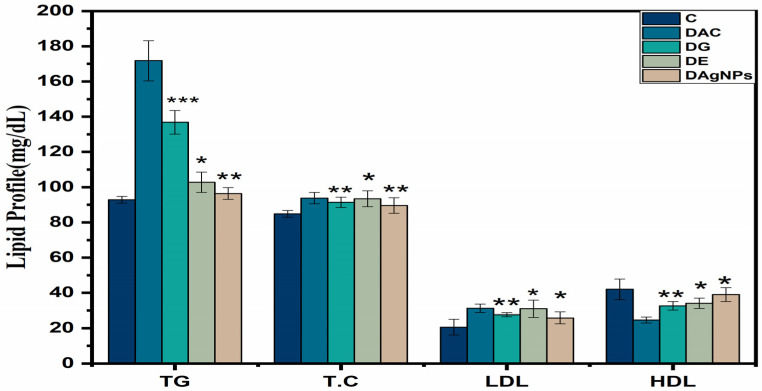
Effect of methanolic extract and synthesised *P. niveum* AgNPs on lipid profile.

The extract and green synthesised NPs showed pronounced effects on the serological parameters of various treated groups. The levels of cholesterol, HDL-c, LDL-c, and TGs in the DAC group were higher than in the DC group as a result of the weight loss (Figure 10).

**Figure 11:**
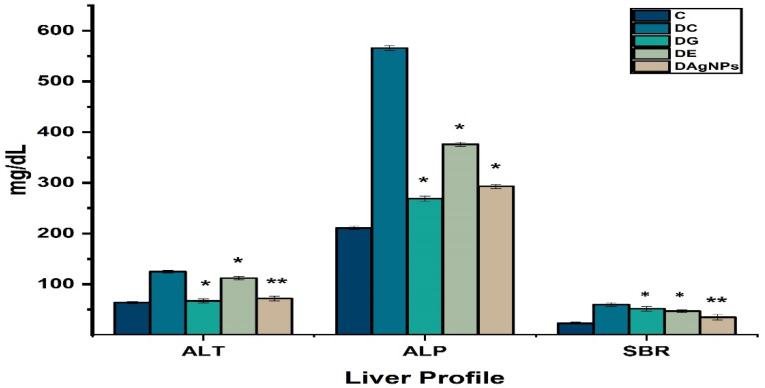
Effect of methanolic extract and synthesised *P. niveum* AgNPs on liver profile.

The results revealed that the liver function test profile is also affected during diabetes mellitus in various treated group. The liver function was altered due to diabetes, with ALT, ALP, and SBR levels increasing in DAC compared to C (Figure 11).

**Figure 12:**
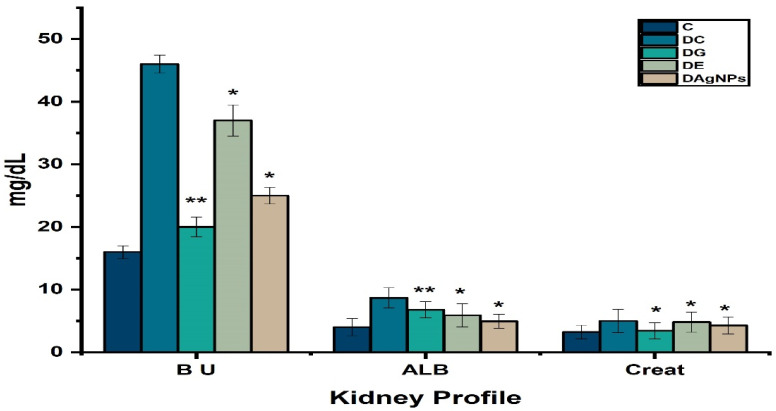
Effect of methanolic extract and synthesised *P. niveum* AgNPs on kidney profile.

In addition, serological tests in the model animal (DAC) have revealed that the levels of blood urea, ALB, and creatinine are higher in the DAC group than in the C group (Figure 12).

The present findings revealed that the kidney function test profile is also affected during diabetes mellitus in various treated group. The majority of urine constituents are higher in DC group animals than in C group animals, according to urine analysis (Table 2).

## 4. Discussion

Phytochemical constituents in the plant samples are known to be biologically active compounds, and they are responsible for different activities, such as antioxidants, antimicrobial, antifungal, and anticancer activities [30,31]. Through different biological mechanisms, all secondary metabolite components displayed antioxidant and antimicrobial properties. Most of the secondary metabolite components were isolated and identified in the polar plant crude extracts [32]. The biochemical screening of the methanolic extract of *P niveum* revealed the presence of phenols, proteins, tannins, flavonoids, alkaloids, quinones, sterols, carbohydrates, amino acids, and terpenoids.

Therefore, the detected different bioactive compounds may be responsible for the antioxidant and antibacterial activities. Several reports are available on flavonoid groups which exhibit high potential biological activities, such as antioxidant, anti-inflammatory, antimicrobial, anticancer, and anti-allergic reactions [33,34,35,36]. Tannins and their derivatives are phenolic compounds considered to be primary antioxidants or free radical scavengers [37,38,39,40,41].

The different parts of plant extracts are eco-friendly, economical, and safe for the synthesis of nanoparticles. In the present study, an attempt was made to synthesise silver nanoparticles from *P. niveum* whole-plant extract. For the synthesis of silver nanoparticles, standardisation was performed with respect to the addition of extract and pH value. The optimisation of these two parameters was essential, as both had a profound effect on the formation of silver nanoparticles. Zayed et al. [42] also reported the effect of extract amount on AgNP formation.

The colourless solution turned brown, indicating the nanoparticle formation of silver. The characteristic brown colour of silver provided a convenient spectroscopic signature to indicate nanoparticle formation. AgNPs are formed in a matter of minutes to hours, as has been reported for other plant extracts [43]. The UV spectra revealed a maximum absorption peak of 410 nm, and the intensity of absorption increased with time. The increase in intensity could be due to the increasing number of nanoparticles formed as a result of the reduction of silver ions present in the aqueous solution with the help of phytoconstituents present in *P. niveum* extract. Similar results were reported by Pant et al. [44] and Roopan et al. [45].

FTIR has become an important tool in understanding the involvement of functional groups in the relations between metal particles and biomolecules. It is used to search for the chemical composition of the surface of the AgNPs and identify the biomolecules for capping and efficient stabilisation of the metal nanoparticles. There were many functional groups present that may have been responsible for the bio-reduction of Ag+ ions. The flavonoids present in the *P. niveum* extract are powerful reducing agents, which may be suggestive of the formation of silver nanoparticles through the reduction of silver nitrate. However, the probable mechanism is unclear and needs further investigation [46].

XRD analysis proved that silver nanoparticles were crystalline in nature. SEM analyses revealed that *P. niveum* AgNPs were spherical, hexagonal, and irregular in shape. The shape and size of nanoparticles formed vary from plant to plant and part to part, as whole plants and the phyto-constituents, such as alkaloid, flavonoids, tannins, and phenols, present in them at the time of synthesis (Table 1).

The AgNPs from *Annona squamosa* leaf extract were spherical in shape, with an average size ranging from 20 to 100 nm [47,48]. They had spherical nanoparticles with a size ranging from 8 to 90 nm in *Desmodium gangeticum*. The sharp signal peak of silver strongly indicated the reduction of silver ions by *P. niveum* into elemental silver. Metallic silver nanoparticles generally show a typical optical absorption peak of approximately 3.25 keV due to surface plasmon resonance. From the EDX spectrum, it was clear that *P niveum* had a percent yield of 71.46% of AgNPs, as reported in [49].

The control group of rats (normal control group) exhibited normal blood glucose levels, whereas alloxan-induced diabetic rats (diabetic control group) showed considerably greater levels than those of the normal control rats [50]. The occurrence of hyperglycaemic conditions led to uncontrolled glucose control brought on by a change in cellular metabolism in diabetic rats [51,52]. It should be noted that diabetic rats treated with AgNPs find their biochemical parameters return to a normal level, illustrating the beneficial outcome with AgNPs. The AgNPs improved blood glucose levels, whereas *P. niveum* extract administration led to less hypoglycaemic activity in alloxan-induced diabetic rats. The oral administration of AgNPs and plant extract for a period of 21 days produced a significantly greater reduction in blood glucose levels than that of *P. niveum*.

The *P. niveum,* extract, and AgNP-treated diabetic rats had a higher glucose tolerance level than the standard drug DG group, which had a lower glucose tolerance level. The DAgNP-treated animals were shown to have significantly lower blood glucose compared to DC. Among the treated groups, the efficiency rank of compounds in lowering the blood glucose level over a period of 2 h was the DG group, followed by the DE and DAgNPs groups. In comparison to the DC group, therapy with DAgNPs lowers blood glucose levels. The alloxan-induced diabetic rat model has been widely used in several studies [53,54]. In addition, the mode of action of alloxan is well documented [55]. The results obtained for blood glucose concentration in the treated groups indicated a decreased total area under the curve, which was considerably less than the DC group, and these findings are in line with previous research. In the treatment of diabetes, blood glucose concentration is regarded as a regular and key biochemical marker to evaluate the improvement in the disease status [56].

In this study, the level of change in the body weight of experimental rats before and after treatment was compared. Weight loss is one of the major syndromes associated with diabetes, probably due to muscle wasting [57]. In our study, the diabetic-induced rat group (DC) showed significant weight loss.

In T2D, the lipid profile is a critical element that must be maintained since elevated lipid levels increase the risk of cardiovascular infections and are usually seen in patients with poorly managed diabetes. As a result of decreased cholesterol synthesis in diabetes, the amount of cholesterol retained will rise [58]. In addition, the increased activation of fatty acids from adipose tissue leads to a disruption of lipid metabolic processes. When alloxan is administered to healthy rats, it produces hyperlipidaemia, which is evident from the increased lipid content detected in diabetic rats who were given alloxan [59]. Extracts of *P. niveum* and AgNPs had an influence on blood total cholesterol, triglycerides, HDL, and LDL levels. In alloxan-induced diabetic rats, cholesterol, triglycerides, and HDL levels all increased, indicating upregulation. Extended cyclic adenosine monophosphate, which is responsible for lipid production, is clearly the cause of the fluctuating blood lipid levels. Treatment with AgNPs resulted in a significant reduction in cholesterol, triglycerides, and LDL levels, as well as an increase in HDL levels in diabetic rats.

## 5. Conclusions

The AgNPs were successfully obtained from the bio-reduction of AgNO_3_ using *P. niveum* methanol extract. The NPs were appropriately characterised and confirmed using different methods, viz., UV-Vis spectroscopy, XRD, FTIR, Scanning Electron Microscopy (SEM), and EDX analysis. Acute toxicity studies were conducted and did not cause any mortality at the dose level tested (i.e., 2000 mg/kg body weight), and under our observation, the levels were considered safe. Plant-mediated NPs demonstrated anti-diabetic activity significantly by lowering blood glucose levels and body weight in rats on days 1, 7, 14, and 21. The levels of blood serum cholesterol, HDL-c, LDL-c, and TGs were improved by the treatment of NPs. This is the first report demonstrating the toxicity parameters of NPs synthesised using *P. niveum*. Since the NPs could be useful in treating type-2 diabetes, further in vivo pharmacological investigations will clearly elucidate the mechanism of action and help in projecting the efficacy of currently synthesised NPs as a therapeutic target in treating type-2 diabetes.

## Figures and Tables

**Table 1:** Qualitative phytochemical analysis of methanol extract of *Phagnalon niveum*.

Tests	Present (+)	Absent (−)
Phenols	+	
Proteins	+	
Glycosides		-
Tannins	+	
Flavonoids	+	
Alkaloids	+	
Saponins		-
Quinones	+	
Sterols	+	
Moline Test for Carbohydrates	+	
Amino acids	+	
Terpenoides	+	
Coumarins	+	

**Table 2:** Effect of methanol extract and synthesised *P. niveum* and AgNPs on urine analysis.

	LEU	NIT	URO	PRO	PH	BLO	SG	KET	BIL	GLU
C	-	-	-	-	5	-	1	-	-	-
DC	15	+	1	30 ± 0.3	5	++	1.01	15 ± 1.5	1	250 ± 15
DG	15	+	1	30 ± 0.4	6	++	1.05	15 ± 1.6	1	-
DE	-	-	1	15	5.5	+	1.01	15 ± 1.7	1	-
DAgNPs	-	-	1	15	5.3	+	1.05	15 ± 1.9	1	-

## Abbreviations

ALB	Albumin protein
ALP	Alkaline phosphatase
ALT	Alanine aminotransferase
BIL	Bilirubin
BLO	Blood
BU	Blood urea
C	Control
C	Carbon
Ca	Calcium
Cl	Chlorine
Creat	Creatinine
ddH_2_O	Double-distilled water
DC	Diabetic control
DE	Diabetic extract
DG	Diabetic glibenclamide
DAgNPs	Diabetic silver nanoparticles
DPP4	Di peptidyl peptidase enzyme 4
EDX	Energy-dispersive X-ray spectroscopy
FTIR	Fourier Transform Infrared spectroscopy
GLU	Glucose
HDLc	High-density lipoprotein
KET	Ketone
LDLc	Low-density lipoprotein
LEU	Leukocytes
Mg	Magnesium
N	Nitrogen
NaOH	Sodium hydroxide
NIT	Nitrite
O	Oxygen
pH	Power of hydrogen ion
PRO	Protein
SBR	Serum bilirubin
SEM	Scanning Electron Microscopy
SGLT-2	Sodium–glucose transport protein 2
SG	Specific gravity
Si	Silicon
T1D	Type-1 diabetes mellitus
T2D	Type-2 diabetes mellitus
TGs	Tri glycerides
URO	Urobilinogen
UV-Vis	Ultraviolet-Visible
XRD	X-ray diffraction
